# Occurrence, Transmission, and Zoonotic Potential of Chronic Wasting Disease

**DOI:** 10.3201/eid1803.110685

**Published:** 2012-03

**Authors:** Samuel E. Saunders, Shannon L. Bartelt-Hunt, Jason C. Bartz

**Affiliations:** University of Nebraska-Lincoln, Omaha, Nebraska, USA (S.E. Saunders, S.L. Bartelt-Hunt);; Creighton University, Omaha (J.C. Bartz)

**Keywords:** Chronic wasting disease, prions and related diseases, bovine spongiform encephalopathy, BSE, transmissible spongiform encephalopathy, TSE, interspecies transmission, environmental persistence, zoonotic potential, cervids, United States, Canada, zoonoses

## Abstract

Chronic wasting disease (CWD) is a fatal, transmissible prion disease that affects captive and free-ranging deer, elk, and moose. Although the zoonotic potential of CWD is considered low, identification of multiple CWD strains and the potential for agent evolution upon serial passage hinders a definitive conclusion. Surveillance for CWD in free-ranging populations has documented a continual geographic spread of the disease throughout North America. CWD prions are shed from clinically and preclinically affected hosts, and CWD transmission is mediated at least in part by the environment, perhaps by soil. Much remains unknown, including the sites and mechanisms of prion uptake in the naive host. There are no therapeutics or effective eradication measures for CWD-endemic populations. Continued surveillance and research of CWD and its effects on cervid ecosystems is vital for controlling the long-term consequences of this emerging disease.

## Abstract 2

This disease continues to emerge in cervids in the United States and Canada.

Chronic wasting disease (CWD) is an inevitably fatal, infectious neurodegenerative prion disease naturally affecting North American mule deer (*Odocoileus hemionus*), white-tailed deer (*Odocoileus virginianus*), elk (wapiti, *Cervus canadensis*), and moose (*Alces alces*) (*1**,**2*). Other prion diseases, or transmissible spongiform encephalopathies, include bovine spongiform encephalopathy (BSE), scrapie in sheep and goats, and Creutzfeldt-Jakob disease (CJD) in humans (*3*). CWD was identified in the late 1960s and recognized as a spongiform encephalopathy by Williams in 1980 (*1*).

Clinical signs of CWD include weight loss and behavioral changes such as altered stance, pacing, excessive salivation, and hyperexcitability that progress over weeks or months (*1*). The infectious agent of CWD is the abnormally folded prion protein (the prion) designated PrP^Sc^, which is distinguished from the normal cellular prion protein (PrP^c^) by its resistance to proteolysis, propensity for aggregation, and insolubility in detergents (*4*). Misfolded prion (PrP^Sc^) can initiate conversion of PrP^c^ to PrP^Sc^ and replicate through a yet unknown mechanism. The exact role that PrP^Sc^ plays in prion disease remains unclear, but PrP^Sc^ is known to accumulate in the central nervous system (CNS) (*1*).

CWD continues to emerge and spread in free-ranging and captive cervids throughout the United States and Canada. Effective therapeutics or and management practices for animal populations in areas to which CWD is endemic do not currently exist. Long-term effects of CWD on cervid ecosystems remain unclear, but the potential for economic consequences is serious because of the role cervids play in the hunting, tourism, and agricultural industries. Moreover, the zoonotic potential of CWD is uncertain, and exposure to CWD-contaminated meat and material will only increase as the disease continues to spread and the incidence increases in areas to which CWD is endemic. We discuss current CWD prevalence and distribution and broadly review surveillance efforts to date. We also present a detailed conceptual model for transmission of the CWD agent and provide an update on CWD interspecies transmission, strains, and zoonotic potential. In addition, we suggest key research needs that may offer hope of slowing or halting the continued emergence of CWD.

## Prevalence and Surveillance

**Figure 1:**
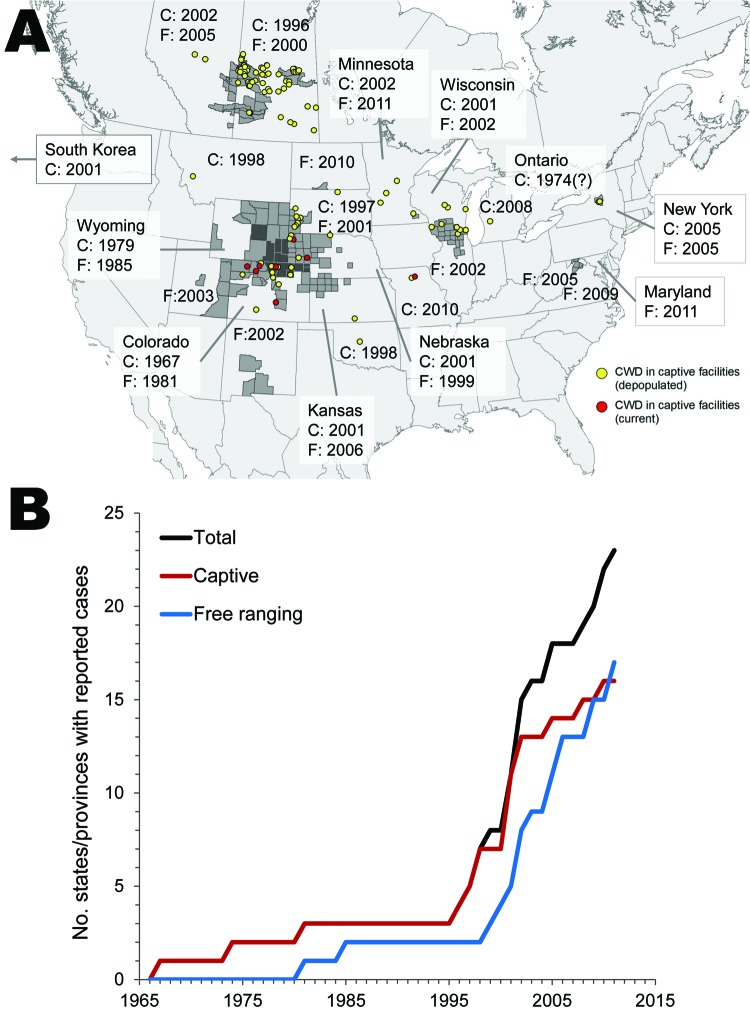
US states and Canadian provinces reporting chronic wasting disease (CWD) cases. A) Year or season CWD was first identified/confirmed in captive (C) or free-ranging (F) cervids. Underlying map shows geographic distribution of CWD (from the US Geological Survey National Wildlife Health Center, updated October 2011, www.nwhc.usgs.gov/disease_information/chronic_wasting_disease/). Light gray shading, current CWD in free-ranging populations; dark gray shading, known distribution of CWD in free-ranging populations before 2000. All locations are approximations based on best available information. B) Cumulative totals of states and provinces that have reported CWD cases in captive or free-ranging cervids. Totals also include South Korea (2001, captive). Many states have reported captive cases for only 1 or 2 years.

Originally recognized only in southeastern Wyoming and northeastern Colorado, USA, CWD was reported in Canada in 1996 and Wisconsin in 2001 and continues to be identified in new geographic locations (Figure 1, panel A). CWD has been identified in free-ranging cervids in 15 US states and 2 Canadian provinces and in ≈100 captive herds in 15 states and provinces and in South Korea (Figure 1, panel B). Except in South Korea, CWD has not been detected outside North America. In most locations reporting CWD cases in free-ranging animals, the disease continues to emerge in wider geographic areas, and prevalence appears to be increasing in many disease-endemic areas. Areas of Wyoming now have an apparent CWD prevalence of near 50% in mule deer, and prevalence in areas of Colorado and Wisconsin is <15% in deer. However, prevalence in many areas remains between 0% and 5% according to reports and data obtained from state and provincial wildlife agencies. Prevalence in elk is lower than in deer but reaches 10% in parts of Wyoming. Known risk factors for CWD include sex and age, and adult male deer show the highest prevalence (*5*). Polymorphisms in the PrP (*PRNP*) gene appear to influence susceptibility in deer and elk (*2**,**6**,**7*), but remain less understood than the strong genetic influences for scrapie.

**Figure 2:**
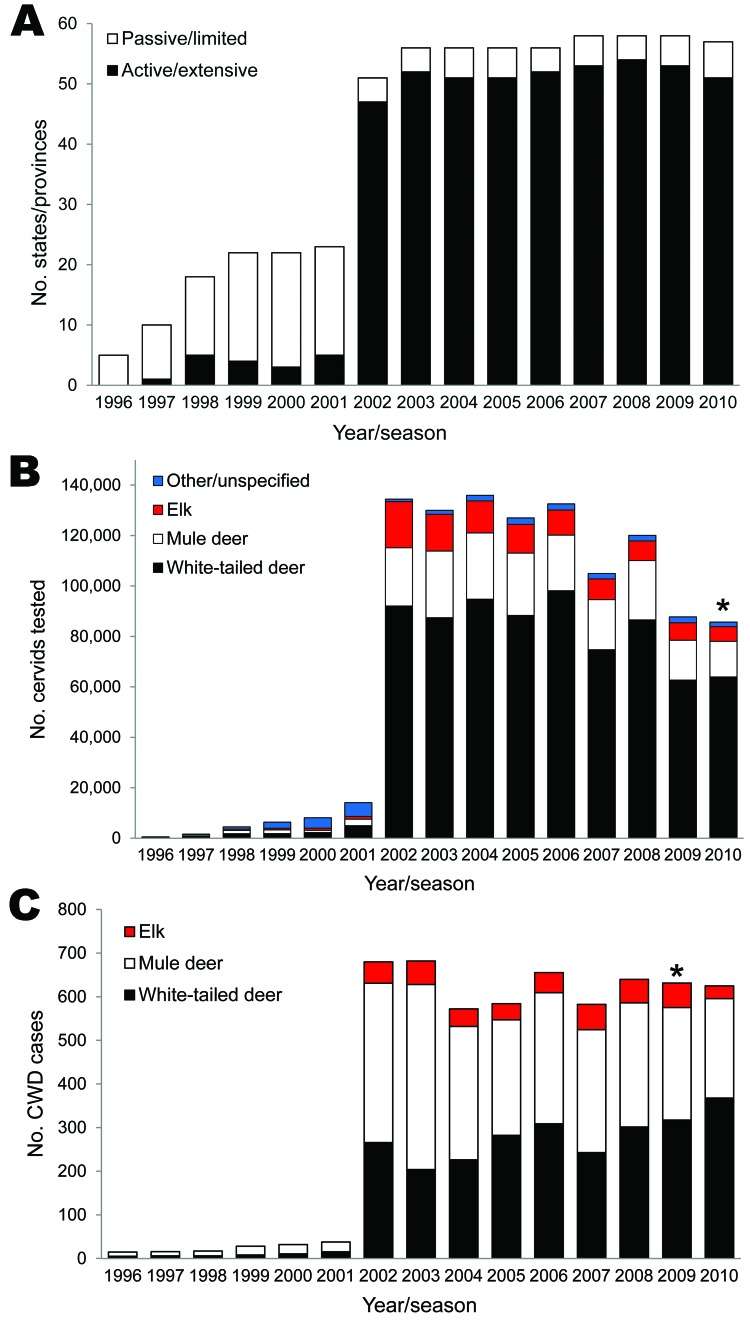
Annual surveillance of free-ranging cervids for chronic wasting disease (CWD). A) Number of US states and Canadian provinces conducting limited or extensive CWD surveillance of free-ranging cervids. B) Number of cervids tested by species each year/season. Other/unspecified includes black-tailed deer, moose, caribou, and data that could not be separated by species. C) Number of CWD-positive cervid samples (CWD cases) by species each year/season. Less than 5 moose were positive. Data were obtained from state and provincial wildlife agencies. Asterisks indicate preliminary or approximated 2010 data.

CWD surveillance programs are now in place in almost all US states and Canadian provinces (Figure 2, panel A). More than 1,060,000 free-ranging cervids have reportedly been tested for CWD (Figure 2, panel B) and ≈6,000 cases have been identified (Figure 2, panel C) according to data from state and provincial wildlife agencies. Following years of limited surveillance in select states and provinces, a nationwide surveillance effort was initiated for the 2002–2003 season, which greatly increased the number of states and provinces performing testing, animals tested, and cases identified (Figure 2). Initial surveillance in most states was generally designed to detect >1 positive animal at a 95% confidence level if the population disease prevalence was >1%, although this goal has not always been achieved.

Many states have now shifted to more targeted surveillance of known disease-endemic areas, areas bordering states reporting cases, or areas surrounding facilities for captive cervids. Samples tested are typically from animals killed by hunters, animals clinically suspected of having CWD, animals killed by vehicles, and targeted sharpshooter kills. Testing of captive cervids is routine in most states and provinces, but varies considerably in scope from mandatory testing of all dead animals to voluntary herd certification programs or mandatory testing of only animals suspected of dying of CWD. A detailed analysis of state and provincial CWD surveillance regimens and disease prevalence is beyond the scope of this report. However, such an analysis would be valuable, not only to evaluate and improve surveillance strategies across the continent (and world) but also to provide insights into spatial and temporal disease dynamics.

Long-term effects of CWD on cervid populations and ecosystems remain unclear as the disease continues to spread and prevalence increases. In captive herds, CWD might persist at high levels and lead to complete herd destruction in the absence of human culling. Epidemiologic modeling suggests the disease could have severe effects on free-ranging deer populations, depending on hunting policies and environmental persistence (*8**,**9*). CWD has been associated with large decreases in free-ranging mule deer populations in an area of high CWD prevalence (Boulder, Colorado, USA) (*5*). In addition, CWD-infected deer are selectively preyed upon by mountain lions (*5*), and may also be more vulnerable to vehicle collisions (*10*). Long-term effects of the disease may vary considerably geographically, not only because of local hunting policies, predator populations, and human density (e.g., vehicular collisions) but also because of local environmental factors such as soil type (*11*) and local cervid population factors, such as genetics and movement patterns (S.E. Saunders, unpub. data).

## Transmission and Role of the Environment

### Horizontal Transmission and Agent Shedding

**Figure 3:**
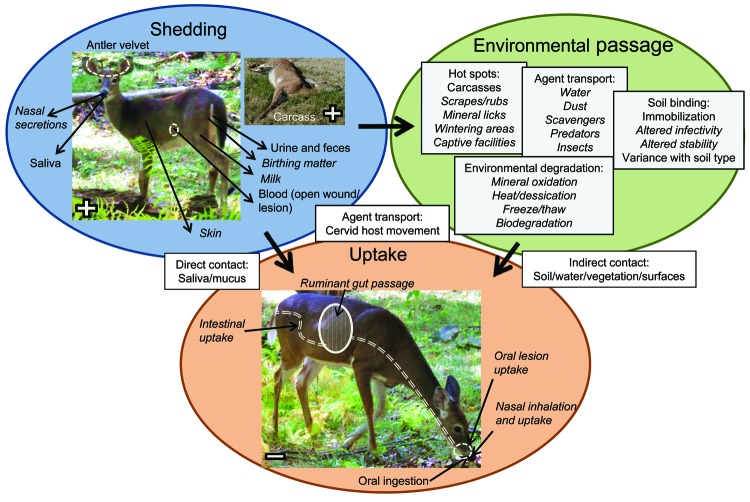
Conceptual model of horizontal transmission of chronic wasting disease (CWD). Items in *italics* are poorly studied or unknown in cervid CWD.

Horizontal transmission of the agent causing CWD is a major mechanism of natural transmission (Figure 3), and maternal transmission is not necessary for disease transmission (*1*). Oral inoculation is an effective route of CWD agent transmission (*1*). Oral lesions facilitate CWD agent transmission in transgenic mice expressing cervid PrP^c^ (*12*). Nasal inoculation is also an effective route of transmission in transgenic mice expressing cervid PrP^c^ (*13*). However, nasal infection and the effect of oral lesions on infection have not yet been evaluated for cervids. Overall, the natural routes and mechanisms of CWD prion uptake are incompletely described.

The CWD agent is shed from infected hosts in urine, feces, saliva, blood, and antler velvet (Figure 3) and can occur in preclinical and clinically affected animals (*14*). CWD prions are also present nearly ubiquitously throughout a diseased host, including skeletal muscle; cardiac muscle; fat; a wide range of glands, organs, and peripheral nervous tissue; and in the highest concentrations in the CNS (*2**,**15*). Thus, CWD prions will enter the environment through shedding from diseased and deaths animals (carcasses). Although quantification of infectious CWD titers in excreta and tissue is challenging, the total titer shed from an infected animal during its lifespan may be approximately equal to the total titer contained in an infected carcass (*16*).

### Indirect Environmental Transmission

Environmental transmission of the CWD agent was reported in studies demonstrating that an infected deer carcass left in a pasture for 2 years could transmit the agent to immunologically naive deer (*17*). Exposure of naive deer to pasture previously inhabited by an infected deer also led to CWD transmission, as did cohabitation of naive and infected deer (*17*). Naive deer exposed to water, feed buckets, and bedding used by CWD-infected deer contracted the disease (*18*).

Epidemiologic modeling suggests that indirect environmental routes of CWD transmission also play a major role in transmission (*8*). Environmental transmission of scrapie is well documented, and scrapie prions may remain infectious after years in the environment (*19**,**20*; S.E. Saunders, unpub. data). Nevertheless, environmental transmission of scrapie may be less efficient than transmission by direct contact (*19*). Conversely, the relative efficiency of CWD transmission by direct contact versus indirect, environmental routes remains unclear, but evidence suggests environmental transmission may be a major mechanism (*8*). The proportion of transmission by direct versus indirect routes may vary not only between captive and free-ranging cervid populations, but also among cervid species and free-ranging habitats and ecosystems. Transmission dynamics may also vary over time as CWD prevalence and ecosystem residence times continue to increase (*8*).

If the environment serves as a reservoir of CWD infectivity, hot spots of concentrated prion infectivity could be formed at areas of communal activity where shedding occurs (Figure 3) (*12*). Animal mortality sites, where highly infectious CNS matter would enter the environment, could also be hot spots (*21*). In a study of deer carcass decomposition in Wisconsin, carcasses persisted for 18–101 days depending on the season, and were visited by deer (*22*). In addition, cervid carcasses are visited by numerous scavenger species, such as raccoons, opossums, coyotes, vultures, and crows, which could consume and transport CWD-infected tissue and increase CWD spread (*21**,**22*). Thus, there is the potential for CWD to spread from sites of animal deaths. Predators may also contribute to spread of the CWD agent and transmission (*5*), as could transport by surface water (*23*) or insect vectors. Natural migration and dispersion of cervids is also a likely mechanism of geographic spread of CWD (*24*).

Given that cervids habitually ingest considerable amounts of soil, soil has been hypothesized to play a key role in CWD transmission (Figure 3) (*11**,**20*; S.E. Saunders et al., unpub. data). Inhalation of dust-bound CWD prions may also represent a route of transmission. It is known that CWD prions can bind to a range of soils and soil minerals (*25**,**26*) and retain the ability to replicate (*27*). In addition, rodent prions retain or gain infectivity when bound to soil and soil minerals (*20**,**27*; S.E. Saunders et al., unpub. data). Prion fate and transmission in soil has been recently reviewed (*20*). Although the potential for CWD transmission by soil and soil reservoirs is considerable, this transmission remains to be directly evaluated with cervids.

## CWD Zoonotic Potential, Species Barriers, and Strains

### Current Understanding of the CWD Species Barrier

Strong evidence of zoonotic transmission of BSE to humans has led to concerns about zoonotic transmission of CWD (*2**,**3*). As noted above, CWD prions are present nearly ubiquitously throughout diseased hosts, including in muscle, fat, various glands and organs, antler velvet, and peripheral and CNS tissue (*2**,**14**,**15*). Thus, the potential for human exposure to CWD by handling and consumption of infectious cervid material is substantial and increases with increased disease prevalence.

Interspecies transmission of prion diseases often yields a species-barrier effect, in which transmission is less efficient compared with intraspecies transmission, as shown by lower attack rates and extended incubation periods (*3**,**28*). The species barrier effect is associated with minor differences in PrP^c^ sequence and structure between the host and target species (*3*). Prion strain (discussed below) and route of inoculation also affect the species barrier (*3**,**28*). For instance, interspecies transmission by intracerebral inoculation is often possible but oral challenge is completely ineffective (*29*).

Most epidemiologic studies and experimental work have suggested that the potential for CWD transmission to humans is low, and such transmission has not been documented through ongoing surveillance (*2**,**3*). In vitro prion replication assays report a relatively low efficiency of CWD PrP^Sc^-directed conversion of human PrP^c^ to PrP^Sc^ (*30*), and transgenic mice overexpressing human PrP^c^ are resistant to CWD infection (*31*); these findings indicate low zoonotic potential. However, squirrel monkeys are susceptible to CWD by intracerebral and oral inoculation (*32*). Cynomolgus macaques, which are evolutionarily closer to humans than squirrel monkeys, are resistant to CWD infection (*32*). Regardless, the finding that a primate is orally susceptible to CWD is of concern.

Interspecies transmission of CWD to noncervids has not been observed under natural conditions. CWD infection of carcass scavengers such as raccoons, opossums, and coyotes was not observed in a recent study in Wisconsin (*22*). In addition, natural transmission of CWD to cattle has not been observed in experimentally controlled natural exposure studies or targeted surveillance (*2*). However, CWD has been experimentally transmitted to cattle, sheep, goats, mink, ferrets, voles, and mice by intracerebral inoculation (*2**,**29**,**33*).

CWD is likely transmitted among mule, white-tailed deer, and elk without a major species barrier (*1*), and other members of the cervid family, including reindeer, caribou, and other species of deer worldwide, may be vulnerable to CWD infection. Black-tailed deer (a subspecies of mule deer) and European red deer (*Cervus elaphus*) are susceptible to CWD by natural routes of infection (*1**,**34*). Fallow deer (*Dama dama*) are susceptible to CWD by intracerebral inoculation (*35*). Continued study of CWD susceptibility in other cervids is of considerable interest.

### Reasons for Caution

There are several reasons for caution with respect to zoonotic and interspecies CWD transmission. First, there is strong evidence that distinct CWD strains exist (*36*). Prion strains are distinguished by varied incubation periods, clinical symptoms, PrP^Sc^ conformations, and CNS PrP^Sc^ depositions (*3**,**32*). Strains have been identified in other natural prion diseases, including scrapie, BSE, and CJD (*3*). Intraspecies and interspecies transmission of prions from CWD-positive deer and elk isolates resulted in identification of >2 strains of CWD in rodent models (*36*), indicating that CWD strains likely exist in cervids. However, nothing is currently known about natural distribution and prevalence of CWD strains. Currently, host range and pathogenicity vary with prion strain (*28**,**37*). Therefore, zoonotic potential of CWD may also vary with CWD strain. In addition, diversity in host (cervid) and target (e.g., human) genotypes further complicates definitive findings of zoonotic and interspecies transmission potentials of CWD.

Intraspecies and interspecies passage of the CWD agent may also increase the risk for zoonotic CWD transmission. The CWD prion agent is undergoing serial passage naturally as the disease continues to emerge. In vitro and in vivo intraspecies transmission of the CWD agent yields PrP^Sc^ with an increased capacity to convert human PrP^c^ to PrP^Sc^ (*30*). Interspecies prion transmission can alter CWD host range (*38*) and yield multiple novel prion strains (*3**,**28*). The potential for interspecies CWD transmission (by cohabitating mammals) will only increase as the disease spreads and CWD prions continue to be shed into the environment. This environmental passage itself may alter CWD prions or exert selective pressures on CWD strain mixtures by interactions with soil, which are known to vary with prion strain (*25*), or exposure to environmental or gut degradation.

Given that prion disease in humans can be difficult to diagnose and the asymptomatic incubation period can last decades, continued research, epidemiologic surveillance, and caution in handling risky material remain prudent as CWD continues to spread and the opportunity for interspecies transmission increases. Otherwise, similar to what occurred in the United Kingdom after detection of variant CJD and its subsequent link to BSE, years of prevention could be lost if zoonotic transmission of CWD is subsequently identified,

## Management Policies

CWD will likely continue to emerge in North America. Given the current extent of CWD and the lack of an effective therapeutic, complete eradication is currently not feasible. As more is learned about disease transmission, it may be possible to manage the prevalence in CWD-endemic areas through hunting policies (*9*). However, long exposures of the environment to CWD prions may create strong environmental reservoirs of CWD capable of efficient transmission, which could sustain or heighten disease incidence (Figure 3) (*8*; S.E. Saunders et al., unpub. data).

Ostensible elimination of CWD in free-ranging cervids has been achieved in only 1 state (New York). After an intensive depopulation and surveillance effort, only 2 free-ranging deer tested were positive for CWD in New York. A similar depopulation and surveillance effort was recently conducted in Minnesota, where only 1 free-ranging deer tested was positive for CWD. Success of the effort in Minnesota and the experience in New York offer hope that new isolated CWD outbreaks can be contained and eliminated by immediate depopulation efforts. However, environmental reservoirs or unknown disease foci may hinder such efforts, and attempts to eliminate CWD in other states in addition to New York have failed. Most notably, an extensive culling effort in Wisconsin that was initiated after CWD detection in 3 free-ranging deer was most likely unsuccessful because the disease was long established in the deer population and environment (*8**,**9*).

Controlling the spread of CWD, especially by human action, is a more attainable goal than eradication. Human movement of cervids has likely led to spread of CWD in facilities for captive animals, which has most likely contributed to establishment of new disease foci in free-ranging populations (Figure 1, panel A). Thus, restrictions on human movement of cervids from disease-endemic areas or herds continue to be warranted. Anthropogenic factors that increase cervid congregation such as baiting and feeding should also be restricted to reduce CWD transmission. Appropriate disposal of carcasses of animals with suspected CWD is necessary to limit environmental contamination (*20*), and attractive onsite disposal options such as composting and burial require further investigation to determine contamination risks. The best options for lowering the risk for recurrence in facilities for captive animals with outbreaks are complete depopulation, stringent exclusion of free-ranging cervids, and disinfection of all exposed surfaces. However, even the most extensive decontamination measures may not be sufficient to eliminate the risk for disease recurrence (*20*; S.E. Saunders et al. unpub. data)

## Research Needs

The influence of environmental factors, such as local climate and habitat characteristics (e.g., vegetation and soil type), on CWD incidence has not been assessed in detail (S.E. Saunders et al., unpub. data). Epidemiologic comparisons of well-established CWD-endemic populations/habitats and newly exposed populations/habitats could yield insights on transmission dynamics. Detection and quantification of environmental CWD prions would be a key step in defining the role of indirect, environmental exposure routes in CWD transmission. Although CWD PrP^Sc^ was detected in a river water sample from an area in Colorado with endemic CWD by using protein misfolding cyclic amplification, the amount detected was below the limit of transgenic mouse bioassay detection, which complicated interpretation of data (*23*).

If environmental reservoirs were implicated in CWD transmission, it may be possible to target these reservoirs for disinfection with a topical enzymatic solution (*26*) or another yet untested treatment and thereby greatly reduce disease incidence. However, cervid density, behavior, and movement may be more significant factors in CWD transmission regardless of the environment. However, such factors also require more investigation. In either case, additional research is needed to determine the natural routes of exposure and agent uptake (Figure 3). CWD prion shedding from cervid birthing matter, milk, nasal secretions, and nonantler skin also warrants investigation because such shedding has been observed with other noncervid prion-infected animals (Figure 3) (*14*).

CWD research tools have been used to make major advances in the past 5 years. Transgenic mouse models of CWD are now invaluable tools for studying CWD infectivity and strains (*2**,**12**,**13**,**15**,**16**,**23**,**30**,**36*), and protein misfolding cyclic amplification has been used effectively for CWD detection, replication, and interspecies studies (*14**,**23**,**27**,**30*). A CWD-susceptible cell culture line is now available (*39*). Continued use of captive cervids in CWD research remains critical to understanding the disease in its natural hosts. Recent advances in premortem detection techniques, including excreta testing (*14*) and rectal biopsy (*40*), may lead to more reliable and noninvasive surveillance programs and enhance experimental capabilities.

## Future CWD Surveillance

The origin of CWD is unknown but may have been either a spontaneous occurrence or caused by interspecies transmission of scrapie or another prion agent. However, because scrapie cases have been reported globally in sheep-farming countries, the potential exits for CWD to occur globally. To our knowledge, CWD surveillance outside the United States and Canada has been largely or completely confined to the industrialized countries of Europe and Asia and has not approached the extensiveness of US and Canadian efforts (Figure 2). Even within North America, surveillance of some cervids, such as caribou, has been limited, and continued enthusiasm for funding and conducting current surveillance programs is uncertain. Given that surveillance efforts are still limited compared with total cervid populations, CWD could be present at low levels in many areas considered free of CWD. At a minimum, targeted surveillance of all cervids within and outside North America should be conducted to understand the true extent of the disease geographically and its host range. This surveillance might be facilitated by more convenient premortem testing methods (*14**,**40*).

## Conclusions

Much remains unknown about prion diseases and CWD in particular, especially about CWD strains (which may have varied zoonotic potentials) and the long-term effects of CWD on cervid ecosystems. CWD prevalence and geographic range appear likely to continue to increase. Moreover, the disease is inevitably fatal, and no effective therapeutic measures are presently available. As such, it would seem wise to continue research and surveillance of CWD to elucidate the details of its transmission, pathogenesis, and continued emergence in cervid populations in hopes that strategies for mitigating its negative effects on humans and cervid ecosystems can be identified.
